# Dietary Pyrroloquinoline Quinone Addition Alleviated Weaning Stress via Modulation of Gut Microbiota and Metabolic Profiles in Weaned Piglets

**DOI:** 10.3390/ani15172543

**Published:** 2025-08-29

**Authors:** Haocheng Xu, Xiuxi Wang, Wenwen Peng, Yashi Hu, Yangyi Xu, Xiao Xiao, Bing Dai, Ruiqiang Zhang, Yifan Zhong, Caimei Yang

**Affiliations:** College of Animal Science and Technology, College of Veterinary Medicine, Zhejiang Agricultural and Forestry University, Hangzhou 311300, China; haochengxu03@foxmail.com (H.X.); 17816737513@163.com (X.W.); penwenwen2022@163.com (W.P.); 15536689099@163.com (Y.H.); 19906839385@163.com (Y.X.); 17367078635@163.com (X.X.); daibing2008@126.com (B.D.); zrq1034@163.com (R.Z.)

**Keywords:** additive, piglet, growth performance, gut microbiota, metabolism

## Abstract

Weaning stress in piglets severely restricts swine production efficiency due to growth retardation, immune suppression, and intestinal dysfunction. This study investigated the effects of dietary pyrroloquinoline quinone (PQQ) on 36 weaned piglets (22 ± 1 days old) allocated to six groups (0, 1, 2, 4, 8 and 16 mg/kg PQQ) for 28 days. Results showed that 4–8 mg/kg PQQ improved average daily gain and feed conversion ratio (*p* < 0.05), enhanced serum immunoglobulin (IgA, IgG) and antioxidant enzyme (T-AOC, SOD, GSH-Px) levels, and reduced inflammatory cytokines (TNF-α, IL-1β, IL-6) (*p* < 0.05). PQQ modulated gut microbiota, increasing *Lactobacillus* and *Bifidobacterium*, and elevated short-chain fatty acid production (*p* < 0.05). Metabolomic analysis revealed upregulated tricarboxylic acid (TCA) cycle intermediates (citric acid, isocitric acid and malic acid), indicating improved mitochondrial function (*p* < 0.05). Overall, 4 mg/kg PQQ optimally alleviates weaning stress by enhancing immunity, gut health, and energy metabolism, offering a promising strategy for piglet nutrition.

## Simple Summary

Weaning stress in piglets is a critical constraint to swine production efficiency, while pyrroloquinoline quinone (PQQ) shows promise as a novel feed additive for stress mitigation. In this study, 216 weaned piglets were fed diets containing 0–16 mg/kg PQQ for 28 days, and results showed that 4–8 mg/kg PQQ optimally improved average daily gain and feed conversion ratio (*p* < 0.05). Addition enhanced serum IgA, IgG, and antioxidant enzymes (T-AOC, SOD, GSH-Px), indicating improved immunity and antioxidant capacity (*p* < 0.05). PQQ also promoted gut health by increasing *Lactobacillus*, *Bifidobacterium*, and short-chain fatty acids (*p* < 0.05), while metabolomic analysis revealed upregulated TCA cycle intermediates (citric, isocitric, malic acids) for better mitochondrial function (*p* < 0.05). Overall, PQQ effectively boosts growth, immunity, and gut health via microbiota and metabolism modulation.

## 1. Introduction

In the growth process of pigs, the weaning period represents the second peak of mortality in the life cycle of piglets [1]. During this period, piglets undergo a series of drastic changes, including environmental shifts, alterations in diet structure, and separation from their dams. These changes challenge the physiological state of piglets, particularly their immune function and intestinal barrier integrity. As a result, piglets often experience slower growth, a high incidence of diarrhea, and even death [2], which pose significant barriers to the pig industry. Therefore, improving weaning stress resilience in piglets and enhancing their survival rate have become major challenges facing the pig industry.

Pyrroloquinoline quinone (PQQ) is a novel quinone-containing, non-covalently bound redox coenzyme found in bacteria. It participates in redox reactions by donating or receiving electrons in the electron transport chain [3]. PQQ exists in three forms, including quinones (oxidized), semi-quinones, and hydroquinones (reduced), depending on the electron-binding conditions in organisms. These forms can interconvert based on the availability of electrons and protons, making PQQ reversible in its reactions [4]. This unique characteristic endows PQQ with a wide range of physiological functions in animals. The high REDOX potential of PQQ confers it with excellent antioxidant properties [5]. Studies have shown that PQQ can scavenge reactive oxygen species (ROS) produced by porcine small intestine epithelial cells [6]. Additionally, PQQ enhances the activity of antioxidant enzymes such as glutathione peroxidase (GSH-Px) and superoxide dismutase (SOD) in weaned piglets [7]. This effect is attributed to PQQ’s ability to bind with REDOX enzymes, altering their conformation and thereby facilitating the elimination of ROS [5].

PQQ also plays a crucial role in mitochondrial health. It removes free radicals generated during mitochondrial energy metabolism, protecting mitochondria from oxidative damage and improving their function. PQQ further promotes mitochondrial biogenesis. For instance, it has been shown to alleviate mitochondrial loss and pathological changes in the brains of Parkinson’s disease mice by activating the AMP-activated protein kinase (AMPK) signaling pathway [8]. Similarly, PQQ addition in pregnant and lactating mice enhances mitochondrial function and lipid oxidation capacity in their offspring, preventing fatty liver development [9]. Beyond its antioxidant and mitochondrial benefits, PQQ promotes growth in young animals. The incorporation of PQQ into the diet significantly augments the production performance and carcass yield in broiler chickens [10]. Recent research has indicated that the administration of PQQ can enhance the physiological status of piglets during the weaning period and facilitate their growth and development [11]. Specifically, the inclusion of PQQ in feed has been shown to enhance growth performance and reduce diarrhea incidence by improving intestinal morphology, tight junction function, and antioxidant levels [12]. Additionally, dietary PQQ addition has been found to mitigate jejunal mucosal inflammation in piglets by inhibiting the NF-κB pathway and regulating colonic microbiota imbalance following an *E. coli* K88 challenge [6]. Overall, PQQ’s multifaceted physiological functions highlight its potential as a valuable nutritional additive in animal husbandry and health. Despite these findings, current research on PQQ in weaned piglets is limited by a narrow dose range and the unclear mechanism of its action. This limitation restricts the broader application of PQQ in piglet nutrition. Therefore, the objective of this study is to determine the optimal dose of PQQ for weaned piglets and to identify its mechanism of action. This research aims to provide a theoretical basis and reference for the practical application of PQQ in weaned piglets.

## 2. Materials and Methods

### 2.1. Animal Ethics

All the studies have been reviewed and approved by the Experimental Animal Ethics Committee of Zhejiang A&F University (EAEC-ZAFU). Approval Number: ZAFUAC2023062.

### 2.2. Animals and Experimental Design

A total of 216 weaned DLY piglets aged 22 ± 1 days, with similar body weights and good health, were selected for the study. The piglets were distributed by gender (half male and half female) and randomly assigned to six treatment groups. Each treatment had 6 replicated pens with 6 pigs per pen. The control group was fed a basal diet without antibiotics. The five groups were fed the same basal diet to which PQQ was added at concentrations of 1, 2, 4, 8, and 16 mg/kg, respectively. The PQQ was provided by Zhejiang Vegamax Biotechnology Co., Ltd., (Hangzhou, China) with Pyrroloquinoline Quinone Disodium Content ≥ 98.0% (based on C_14_H_4_N_2_Na_2_O_8_ dry basis). Piglets were ear-tagged, weighed, and granted ad libitum access to feed and water throughout the 28-day trial period. Daily feed intake, mortality rate, and PQQ addition dosage were meticulously recorded. The composition and nutritional level of the feed used in this study are presented in Table 1. To detect Organic Matter (OM) in feed, samples were combusted in a muffle furnace at 550 °C to determine ash, OM = 100% − ash% [13]. The contents of crude protein (CP) in the feed were measured using the Kjeldahl method [14]. The digestible energy (DE) content in feed was calculated in accordance with the China National Standard NY/T 65-2004 [15]. The determination of neutral detergent fiber (NDF) in feed was carried out by the Van Soest detergent fiber analysis method with 3% sodium dodecyl sulfate (SDS) [16]. The determination of acid detergent fiber (ADF) in feedstuffs was performed using the Van Soest detergent fiber analysis method with 2% cetyltrimethylammonium bromide (CTAB) [16]. Digestible amino acids (AA) were determined by the acid hydrolysis method (GB/T 18246-2019) [17]. According to China National Standard (GB/T 6436-2018) [18], EDTA disodium complexometric titration was used to determine the contents of calcium (Ca). The contents of total phosphorus (TP) were measured by spectrophotometry in accordance with China National Standard (GB/T 6437-2018) [19]. The basal diet was formulated in accordance with the nutrient specifications recommended by the National Research Council [20].

### 2.3. Growth Performance

Individual piglets were weighed at 8:00 a.m. and again 12 h after fasting at Day 0, Day 14 and Day 28 of the study. On days 14 and 28, the weight of residual feed for each group of piglets was measured. The feed intake of each group was calculated by subtracting the weight of residual feed from the total feed provided. Mortality and morbidity rate were recorded to evaluate the health status of each group. The growth performance indicators, including average daily gain (ADG), average daily feed intake (ADFI), and feed-to-gain ratio (F: G), were calculated based on these measurements.

F: G = ADG/ADFI.

### 2.4. Sample Collection and Processing

At the termination of the experiment, one piglet (with a body weight approximating the average body weight of the pen) was randomly selected from each replicate pen of each treatment group for slaughter, resulting in a total of 36 piglets. Piglets were euthanized by intracardiac administration of sodium pentobarbital (50 mg/kg body weight) followed by jugular exsanguination, after which tissue sampling was performed.

### 2.5. Hematological Testing

At 14 Day and 28 Day, blood samples were collected from the anterior vena cava of piglets in each replicate of every group. These samples were analyzed for physiological parameters related to the test substance. The following indicators were detected for all groups: White blood cell (WBC), red blood cell (RBC), hemoglobin (HGB), hematocrit (HCT), mean corpuscular volume (MCV), mean corpuscular hemoglobin (MCH), mean corpuscular hemoglobin concentration (MCHC), red blood cell distribution width (RDW), platelet (PLT). The control group, 4 mg/kg group, and 8 mg/kg group were subjected to the detection of the following indicators: Immunoglobulin A (IgA), immunoglobulin G (IgG), immunoglobulin M (IgM), total antioxidant capacity (T-AOC), superoxide dismutase (SOD), glutathione peroxidase (GSH-Px), tumor necrosis factor-α (TNF-α), interleukin-1β (IL-1β), interleukin-6 (IL-6).

### 2.6. Histopathological

At the end of the experiment, a systematic histopathological examination was performed on the duodenum, jejunum and ileum of the control group, the 4 mg/kg and the 8 mg/kg dose groups. The intestinal tissues were subjected to dehydration and clearing, followed by embedding in paraffin. Subsequently, the tissues were sectioned using a microtome to produce paraffin-embedded sections. The sections were stained with hematoxylin and eosin (HE) for histological evaluation. Images were captured using a Nikon optical microscope (Tokyo, Japan).

The villus length and crypt depth of the duodenum, jejunum, and ileum were observed and measured, and the villus length to crypt depth ratio (V/C) was calculated.

V/C = villus length (μm)/crypt depth (μm).

### 2.7. Short-Chain Fatty Acid

Cecal content samples and feces samples were collected from experimental piglets at the end of the trial. Cecal content samples derived from the control group, 4 mg/kg PQQ group, and 8 mg/kg PQQ group were selected for detection. The samples (0.5 g) were dissolved in ddH_2_O at a mass-to-volume ratio of 1:3. Following centrifugation at 12,000× *g* for 10 min to remove impurities, added 25% metaphosphoric acid at a volume ratio of 5:1, and centrifuged again at 12,000× *g* for 10 min to further remove impurities. The supernatant was filtered through an inorganic phase filter head using a 1 mL syringe and transferred to an injection vial suitable for gas chromatography (GC7890, Agilent Technologies, Santa Clara, CA, USA). Short-Chain Fatty Acid (SCFA) content was analyzed using a gas chromatograph equipped with a HP-FFAP column (Beijing Pumeng Technology Co., Ltd., Beijing, China). SCFA concentrations were directly read from the instrument following calibration.

### 2.8. Gut Microbiota

Cecal contents samples derived from the control group, 4 mg/kg PQQ group, and 8 mg/kg PQQ group were selected for detection. Genomic DNA was extracted from cecal contents using the SDS method. The V3–V4 region of the 16S rRNA gene was amplified by three-step PCR. The primer set 341F and 806R (341 F: 5′- CCTACGGGRSGCAGCAG -3′, 806R: 5′- GGACTACVVGGGTATCTAATC -3′) was applied for the amplification of gene fragment. The amplified products were subjected to agarose gel electrophoresis for quality assessment, followed by equimolar pooling and re-quantification. Purification of the amplicons was performed using a commercial recovery kit. Subsequently, the purified products were sequenced on the MiSeq platform (Illumina, San Diego, CA, USA). The raw sequencing data were processed using QIIME 2 (version 2020.8; (https://qiime2.org, accessed on 15 December 2023)), which included demultiplexing, quality filtering, dereplication, chimera detection, and merging of paired-end reads. Downstream analysis and visualization of the microbiota data, including alpha and beta diversity analyses, were conducted using R software (version 3.3.1).

### 2.9. Metabolomics

Serum contents samples derived from the control group, 4 mg/kg PQQ group, and 8 mg/kg PQQ group were selected for detection. For metabolite extraction, 100 μL of serum was mixed with 500 μL of 80% methanol aqueous solution and extracted on ice for three cycles, each lasting 10 min. The samples were then kept at −20 °C for 30 min and centrifuged at 13,000× *g* for 15 min at 4 °C to isolate the metabolites, which were transferred to an injection vial for analysis. LC-MS/MS analysis was performed by Majorbio Bio-Pharm Technology Co., Ltd. (Shanghai, China) using a UHPLC-Orbitrap Exploris 240 system with an ACQUITY HSS T3 column (100 mm × 2.1 mm i.d., 1.8 μm; Waters, Milford, MA, USA). Mobile phases were solvent A (0.1% formic acid in water: acetonitrile, 2:98, *v*/*v*) and solvent B (0.1% formic acid in acetonitrile), with a flow rate of 0.40 mL/min, column temperature of 40 °C, and injection volume of 5 μL. MS conditions: The UPLC system was coupled to the UHPLC-Orbitrap Exploris 240 mass spectrometer with an ESI source (positive/negative modes). Optimal settings: source temperature 400 °C; sheath gas 40 arb; aux gas 10 arb; ISVF −2800 V (negative) and 3500 V (positive); normalized collision energy 4. MS/MS used 20–40–60 V rolling voltage. Data were acquired in DDA mode over 70–1050 m/z. The R package “ropls” (v1.6.2) was used for PCA, OPLS-DA, and 7-cycle interactive validation to assess model stability. Significantly different metabolites were identified as those with VIP > 1 (from OPLS-DA) and *p* < 0.05 (from Student’s *t*-test). Differential metabolites between groups were mapped to biochemical pathways via KEGG-based metabolic enrichment and pathway analysis, and classified by involved pathways or functions. Enrichment analysis (using Python package “scipy.stats” version 1.7.3) determined if metabolite groups were present in functional nodes (extending single-metabolite to group annotation) to identify key biological pathways related to experimental treatments.

### 2.10. Statistical Analysis

Excel 2021 was used to preliminarily sort out data. The General Linear Model (GLM) procedure within SPSS 16.0 was utilized to conduct analysis of variance. The data of the remaining indicators were analyzed using Gaussian linear mixed model with SPSS for all variables.

Y_ijk_ = μ + F_i_ + I_j_ + R_k_ + e_ijk_

Individuals were considered random effects, and the dietary PQQ level was considered a fixed effect. Data points exhibiting deviations exceeding ±2 standard deviations from the mean were identified as outliers and subsequently excluded from the analysis to ensure robustness. The data were plotted using GraphPad Prism version 8.0 software. And Biorender (Scientific Image and Illustration Software|BioRender version 1.0.0.3) was applied for the illustration of figures. Statistical analysis shows significant differences between different treatments, with *p* < 0.05 indicating significant differences, *p* < 0.01 indicating extremely significant differences, and *p* > 0.05 indicating insignificant differences. Correlation analyses were calculated using Spearman correlation analysis.

## 3. Results

### 3.1. Growth Performance

As indicated in Table 2, the differences in weight at 28 days, ADG from 15 to 28 days and from 0 to 28 days. and F: G from 15 to 28 days and from 0 to 28 days were significant among different treatments (*p* < 0.05). Meanwhile, the weight and F: G of weaned piglets also showed a quadratic correlation with the addition level of PQQ. Specifically, piglets with 4 mg/kg and 8 mg/kg PQQ added (to their diet) exhibited significantly higher weight at 28 days and ADG (both from 15 to 28 days and from 0 to 28 days) (*p* < 0.05). And piglets with 4 mg/kg and 8 mg/kg PQQ added (to their diet) exhibited significantly lower F: G (both from 15 to 28 days and from 0 to 28 days) compared to the control group (*p* < 0.05).

### 3.2. Hematology

As indicated in Table 3, hematology of piglets with PQQ added (to the diet) were within the normal range across all groups, with no significant differences observed among treatments (*p* > 0.05).

### 3.3. Immunity and Antioxidation

As depicted in Table 4, on Day 14, piglets with 4 mg/kg and 8 mg/kg PQQ added (to their diet) exhibited significantly higher serum IgG levels compared to the control group (*p* < 0.05). Additionally, piglets with 4 mg/kg PQQ added (to their diet) showed significant increases in serum levels of T-AOC, and GSH-Px compared to the control group (*p* < 0.05). On Day 28, piglets with 4 mg/kg and 8 mg/kg PQQ added (to their diet) had significantly higher serum IgA levels than the control group (*p* < 0.05). Furthermore, piglets with 4 mg/kg added (to their diet) exhibited a significant elevation in serum T-AOC levels compared to the control group (*p* < 0.05).

### 3.4. Serum Inflammatory Cytokines

Table 4 illustrates that serum levels of the inflammatory cytokines TNF-α, IL-1β, and IL-6 were significantly downregulated in piglets with 4 mg/kg and 8 mg/kg PQQ added (to their diet) compared to the control group (*p* < 0.05) on Day 14. Similarly, serum IL-6 levels were significantly reduced in piglets with 4 mg/kg and 8 mg/kg PQQ added (to their diet) compared to the control group (*p* < 0.05) on Day 28.

### 3.5. Intestinal Morphology

**Figure 1:**
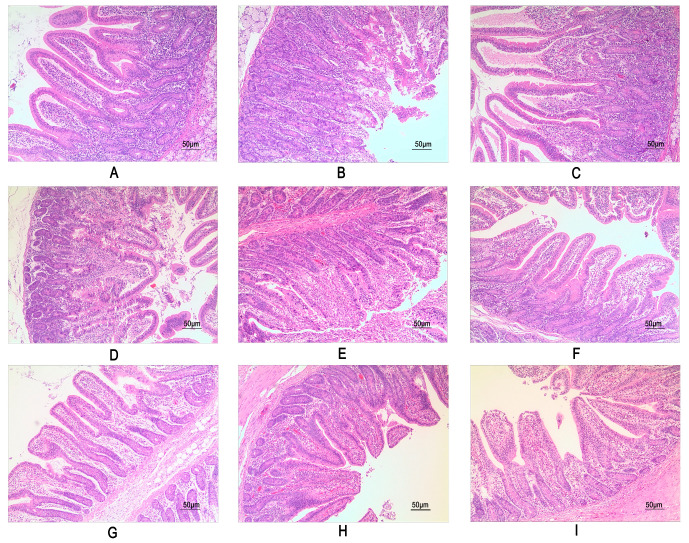
Intestinal morphology of piglets fed graded levels of PQQ. (**A**–**C**) depict the duodenum, jejunum, and ileum of the control group; (**D**–**F**) show the corresponding tissues from weaned piglets with 4 mg/kg PQQ added (to their diet); and (**G**–**I**) show the corresponding tissues from weaned piglets with 8 mg/kg PQQ added (to their diet).

Dietary addition with 4 mg/kg and 8 mg/kg PQQ had no significant effect on the morphological structure of the duodenum, jejunum, and ileum in weaned piglets (Figure 1). Histological examination of HE-stained sections from the control and PQQ groups revealed that the intestinal villi exhibited a regular morphology, characterized by finger-like projections. The villous surface was lined by a uniform layer of columnar epithelial cells, which were arranged in an orderly manner with distinct and clearly demarcated nuclei. As indicated in Table 5, the addition of PQQ exerted no significant influence on the intestinal villus length, crypt depth, or villus-to-crypt ratio (V/C) in the duodenum, jejunum, and ileum of weaned piglets (*p* > 0.05).

### 3.6. Short-Chain Fatty Acid

As shown in Table 6., the fecal concentrations of acetic acid, propionic acid, isobutyric acid, butyric acid, and valeric acid were significantly higher in piglets with 4 mg/kg and 8 mg/kg PQQ added (to their diet) compared to the control group (*p* < 0.05). Additionally, the fecal concentration of isovaleric acid was significantly higher in piglets with 8 mg/kg PQQ added (to their diet) than in those piglets with 4 mg/kg PQQ added (to their diet) or in the control group (*p* < 0.05). In contrast, there was no significant difference in fecal isovaleric acid concentration between the 4 mg/kg PQQ group and the control group (*p* > 0.05).

### 3.7. Gut Microbiota

**Figure 2:**
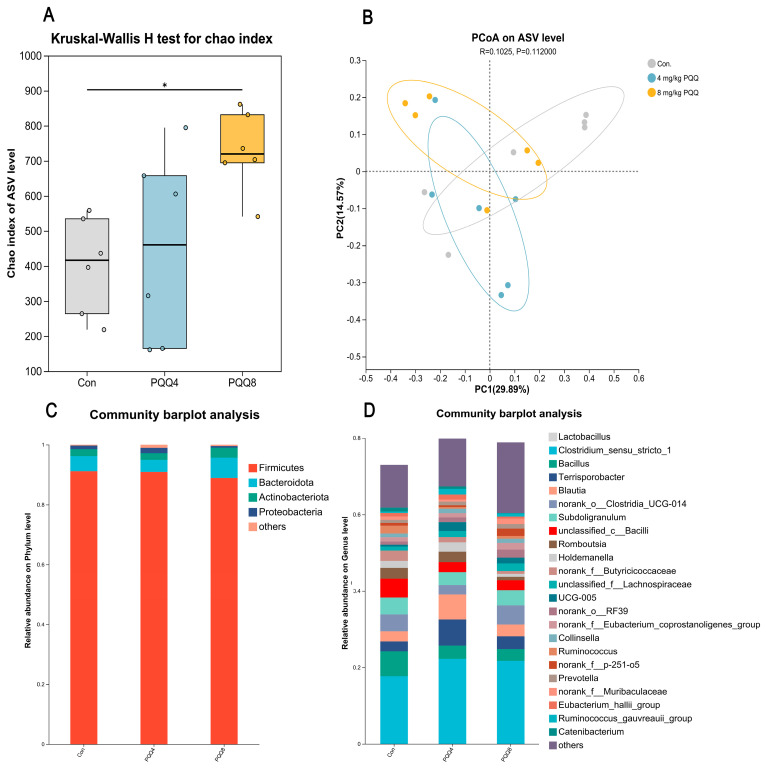
Microbial community diversity and composition analysis in the feces of piglets fed graded levels of PQQ for 28 d (*n* = 6). (**A**) Alpha diversity on ASV level. The *x*-axis represents different experimental groups, and the *y*-axis represents the Chao index of cecal flora at the ASV level in each group. * *p* < 0.05. (**B**) Beta diversity on ASV level. The scales on both axes represent relative distances and hold no inherent quantitative significance. Points differentiated by color correspond to samples from distinct groups; a smaller distance between two sample points indicates a higher similarity in their species composition. (**C**) Relative abundance on phylum level. The *x*-axis represents different experimental groups, and the *y*-axis represents relative abundance on phylum level in each group. (**D**) Relative abundance on genus level. The *x*-axis represents different experimental groups, and the *y*-axis represents relative abundance on genus level in each group. Con = Control Group; PQQ4 = 4 mg/kg PQQ; PQQ8 = 8 mg/kg PQQ.

**Figure 3:**
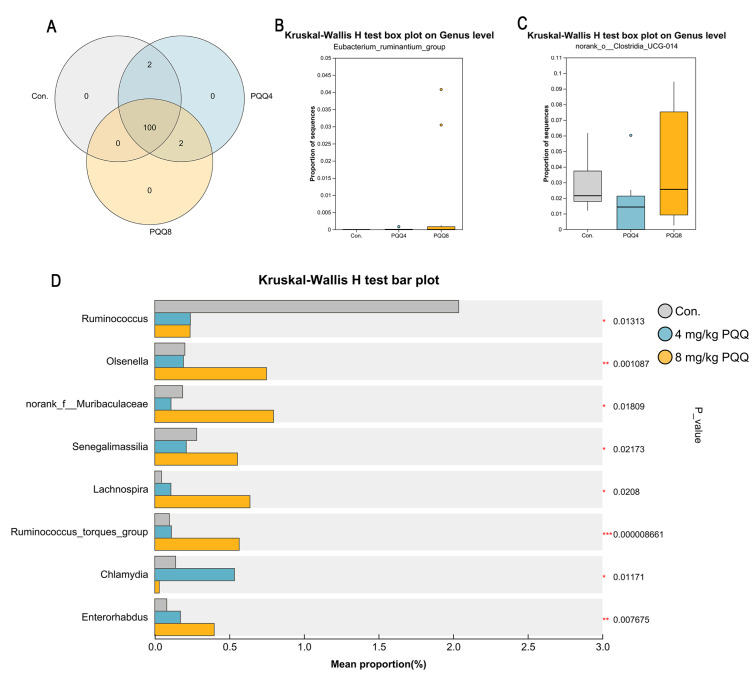
Microbial Species Comparative Analysis in the feces of Piglets fed graded levels of PQQ for 28 d (*n* = 6). (**A**) Species Venn Diagram Analysis on genus level. Different colors correspond to different groups. Overlapping regions denote the genera shared among multiple groups, whereas non-overlapping regions represent the genera uniquely associated with a specific group. The numbers indicate the corresponding count of genera. (**B**) Relative abundance of *Eubacterium_ruminantium_group* in each group. The *x*-axis represents different experimental groups, and the *y*-axis represents relative abundance on genus level in each group. (**C**) Relative abundance of *norank_o_Clostridia_UCG-014* in each group level. The *x*-axis represents different experimental groups, and the *y*-axis represents relative abundance on genus level in each group. (**D**) Multiple-Group Comparative Analysis of Species Differences. The *y*-axis represents the species names on genus levels, while the *x*-axis denotes the percentage values of the abundance of a specific species in each group. Different colors correspond to different groups. The rightmost column indicates *p*-values, with * indicating 0.01 < *p* ≤ 0.05, ** indicating 0.001 < *p* ≤ 0.01, and *** indicating *p* ≤ 0.001. Con = Control Group; PQQ4 = 4 mg/kg PQQ; PQQ8 = 8 mg/kg PQQ.

As depicted in Figure 2A, the Ace index in piglet feces was significantly elevated following dietary addition with 8 mg/kg PQQ (*p* < 0.05). PCoA analysis (Figure 2B) revealed that the fecal microbiota composition of piglets with 4 mg/kg and 8 mg/kg PQQ added (to their diet) was distinct from that of the control group. Figure 2C,D illustrate the fecal microbial community structure at the phylum and genus levels, respectively, based on taxonomic analysis. At the phylum level, the dominant phyla were Firmicutes, Bacteroidota, Proteobacteria, and Actinobacteriota (Figure 2C). At the genus level, the dominant genera included *Lactobacillus, Clostridium_sensu_stricto_1*, *Bacillus*, *Terrisporobacter*, *Blautia*, *norank_o_Clostridia_UCG-014*, *Subdoligranulum*, *unclassified_c_Bacilli* and *Romboutsia*, (Figure 2D). Figure 3 presents the differential analysis of fecal microbial species in weaned piglets fed varying levels of PQQ. The Venn diagram (Figure 3A) shows that, compared to the control group, piglets with PQQ added (to their diet) had only two distinct species at the ASV (Amplicon Sequence Variant) level: *Eubacterium ruminantium group* (Figure 3B) and *norank_o_Clostridia_UCG-014* (Figure 3C). The relative abundance of these taxa did not significantly differ among groups. Figure 3D illustrates genera with significant differences in relative abundance among the groups, listed in descending order of abundance: *Subdoligranulum*, *Escherichia-Shigella*, *Ruminococcus*, *Olsenella*, *norank_f__Muribaculaceae*, *Senegalimassilia*, *Lachnospira*, *Ruminococcus_torques_group*, *Chlamydia*, *and Enterorhabdus*.

**Figure 4:**
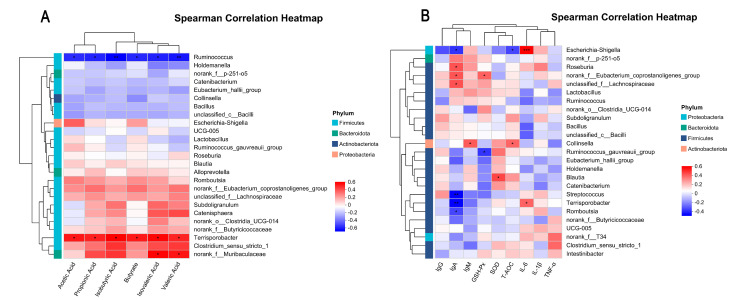
Spearman correlation heatmap (*n* = 6). (**A**) Spearman’s correlation analysis of fecal microbiota and SCFAs in Weaned Piglets, (**B**) Spearman’s correlation analysis of fecal microbiota and immune and inflammatory indicators in weaned piglets. The *X*-axis and *Y*-axis correspond to different factors and genera, respectively, with *R*-values and *p*-values derived from calculations. *R*-values are visualized in distinct colors in the figure; *p*-values less than 0.05 are marked with *. The legend on the right indicates the color intervals corresponding to different R-values. * *p* < 0.05, ** *p* < 0.01, *** *p* < 0.001.

The correlation analysis between fecal microbiota and SCFAs in weaned piglets is presented in Figure 4A. The analysis reveals that, at the genus level, *Terrisporobacter* and *norank_f_Muribaculaceae* are significantly associated with increased SCFAs content in feces, whereas *Ruminococcus* is associated with decreased SCFAs content. The correlation analysis between fecal microbiota and serum immune and inflammatory markers is illustrated in Figure 4B. Specifically, *Roseburia* and *unclassified_f_Lachnospiraceae* exhibit positive correlations with IgA levels. *Norank_f_Eubacterium_coprostanoligenes_group* is positively associated with both IgA and GSH-Px levels. Additionally, *Collinsella* displays a positive correlation with IgM and T-AOC levels, while *Blautia* is positively correlated with SOD levels. In contrast, *Escherichia-Shigella* is negatively correlated with IgA and T-AOC levels but positively correlated with IL-6 levels. *Ruminococcus_gauvreauii_group* demonstrates a negative correlation with GSH-Px levels. Furthermore, *Streptococcus* and *Romboutsia* are negatively associated with IgA levels. *Terrisporobacter* is negatively correlated with IgA levels yet positively correlated with IL-6 levels.

### 3.8. Metabolism

**Figure 5:**
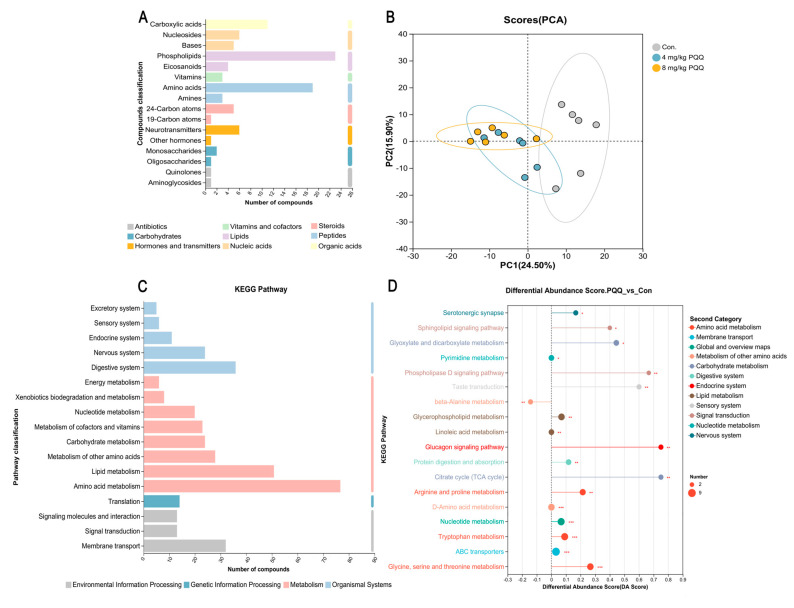
Metabolism in the serum of piglets fed different levels of PQQ for 28 d. (**A**) Bar chart of KEGG compound classification statistics. The *y*-axis represents KEGG compound classifications, and the *x*-axis denotes the number of compounds annotated to each category. The color of the bars indicates the primary classification category to which the compounds belong. (**B**) PCA analysis plot. The scales on both axes represent relative distances and hold no inherent quantitative significance. Points differentiated by color correspond to samples from distinct groups; a smaller distance between two sample points indicates a higher similarity in metabolites composition. (**C**) KEGG pathway statistics chart. The *y*-axis represents the secondary classifications of KEGG metabolic pathways, and the *x*-axis denotes the number of metabolites annotated to each pathway. The color of the bars indicates different categories of metabolic pathways. (**D**) KEGG pathway differential abundance (DA score) analysis plot (*n* = 6). The figure illustrates the analysis of differential metabolic pathways between the PQQ-added group and the control group. The *x*-axis in the figure represents the differential abundance score (DA Score), and the *y*-axis denotes the names of KEGG metabolic pathways. The DA Score reflects the overall changes of all metabolites in a metabolic pathway: a score of 1 indicates that all annotated differential metabolites in the pathway show an upward expression trend, while a score of −1 indicates a downward expression trend of all annotated differential metabolites in the pathway. The length of the line segment corresponds to the absolute value of the DA Score. The size of the dot represents the number of annotated differential metabolites in the pathway, with larger dots indicating a greater number of differential metabolites in that pathway. Dots distributed on the right side of the central axis with longer line segments indicate a stronger tendency of overall upregulation in the pathway; conversely, dots distributed on the left side of the central axis with longer line segments indicate a stronger tendency of overall downregulation in the pathway. * *p* < 0.05, ** *p* < 0.01, *** *p* < 0.001.

The effects of PQQ addition on the serum metabolome of weaned piglets were shown in Figure 5. And 92 metabolites in nine major categories were detected in all samples, with peptides and lipids being the most abundant (Figure 5A). The PCA analysis indicates significant changes in serum metabolites of piglets with 4 mg/kg and 8 mg/kg PQQ added (to their diet) compared to the control group (Figure 5B).

The number of metabolites enriched in KEGG pathways is shown in Figure 5C. The analysis reveals 17 pathways with abundant metabolite species, including amino acid metabolism (77 metabolites), lipid metabolism (51 metabolites), digestive system (36 metabolites), membrane transport (32 metabolites), metabolism of other amino acids (28 metabolites), nervous system (24 metabolites), carbohydrate metabolism (24 metabolites), metabolism of cofactors and vitamins (23 metabolites), nucleotide metabolism (20 metabolites), translation (14 metabolites), signal transduction (13 metabolites), signaling molecules and interaction (13 metabolites), endocrine system (11 metabolites), xenobiotics biodegradation and metabolism (8 metabolites), energy metabolism (6 metabolites), sensory system (6 metabolites), excretory system (5 metabolites). Figure 5D displays the KEGG pathway differential abundance score plot. The top 10 significantly upregulated metabolic pathways, ranked in descending order, are as follows: TCA cycle (DA score = 0.75, *p* < 0.01), glucagon signaling pathway (DA score = 0.75, *p* < 0.01), phospholipase D signaling pathway (DA score = 0.67, *p* < 0.01), taste transduction (DA score = 0.60, *p* < 0.01), Glyoxylate and dicarboxylate metabolism (DA score = 0.44, *p* < 0.05), sphingolipid signaling pathway (DA score = 0.40, *p* < 0.05), glycine, serine and threonine metabolism (DA score = 0.27, *p* < 0.01), neuroactive ligand-receptor interaction (DA score = 0.23, *p* < 0.05), arginine and proline metabolism (DA score = 0.21, *p* < 0.01), central carbon metabolism in cancer (DA score = 0.19, *p* < 0.001).

**Figure 6:**
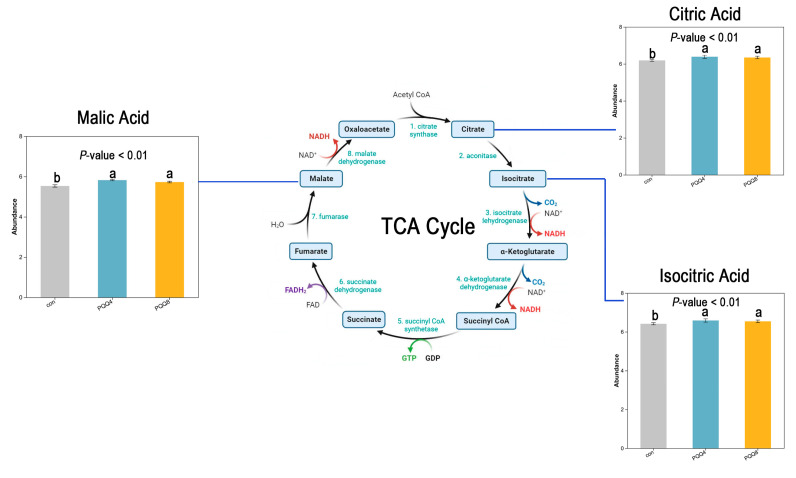
Figure of TCA cycle and the abundance of its intermediate products. The circular line represents the TCA pathway map. The bar charts indicate the metabolites with significant differences in the TCA pathway among different treatment groups. The *x*-axis represents the groups, and the *y*-axis represents the metabolite abundance. a, b, means with no identical letters differ significantly (*p* < 0.01). Con = Control Group; PQQ4 = 4 mg/kg PQQ; PQQ8 = 8 mg/kg PQQ.

TCA cycle (map00020) was identified as the most significantly upregulated pathway. The TCA cycle is a central metabolic pathway involved in energy production and the synthesis of various biomolecules [21]. The analysis showed that compared with the control group, the levels of Citric Acid, Isocitric Acid and Malic Acid in this pathway were significantly increased in the PQQ-added group (*p* < 0.01). (Figure 6).

## 4. Discussion

PQQ is emerging as a novel growth-promoting factor for animals and holds potential as a feed additive [22]. Therefore, adding PQQ to the feed of piglets is a potential intervention strategy to alleviate weaning stress. The study was designed to investigate the effects of different doses of PQQ on weaned piglets. The results demonstrated that addition with 4 mg/kg and 8 mg/kg PQQ significantly enhanced growth performance. These two dosages of PQQ outperformed other dosages tested. To further elucidate the mechanisms underlying these effects, detailed analyses were conducted on the 4 mg/kg and 8 mg/kg dose groups. The findings disclosed the multifaceted impacts of PQQ addition in weaned piglets. It was demonstrated that PQQ addition led to significant improvements in immune function, a notable reduction in inflammatory markers, and substantial alterations in both the composition of gut microbiota and the metabolic profiles of weaned piglets.

Previous research has demonstrated that adding the basal diet of weaned piglets with 3 mg/kg PQQ can effectively reduce F: G and enhance ADG [2]. In the current experiment, the ADG of weaned piglets was significantly elevated by the addition of PQQ. The ADFI of the group added with PQQ did not show a significant increase compared to the control group. These results imply that PQQ exerts its positive impact on growth performance by augmenting feed conversion efficiency rather than through an increase in feed intake. The present study indicates that both 4 mg/kg and 8 mg/kg PQQ exhibit favorable effects in improving the growth performance of piglets. This indicates that the optimal PQQ concentration for improving growth performance in weaned piglets lies between 4 mg/kg and 8 mg/kg. These results are consistent with those reported by Yin. They found that piglet growth performance peaked with 4.5 mg/kg PQQ addition [12]. In terms of cost-effectiveness, 4 mg/kg PQQ offers a lower relative cost when compared to 8 mg/kg PQQ. In addition to the aforementioned analyses, hematological examinations and HE staining of intestinal sections were carried out for the control group and the groups added with 4 mg/kg and 8 mg/kg PQQ. No adverse effects were observed in these assessments. This indicates the tolerance of weaned piglets to PQQ addition. A recent study found that dietary addition of pigs with 75.0 mg/kg PQQ had no adverse effect on their growth performance [11]. This finding indicates that PQQ is well-tolerated and safe for use in pig diets. As a result, it supports the potential of PQQ as a growth-promoting feed additive.

According to the results of this experiment, there is a quadratic correlation between dosage of PQQ added (to the diet) and the growth performance of weaned piglets, among which the effects of the 4 mg/kg and 8 mg/kg dosage groups are the most significant. In view of this, subsequent studies will focus on conducting in-depth investigations into the action mechanisms of 4 mg/kg and 8 mg/kg PQQ on weaned piglets.

Weaning stress in piglets is associated with a cascade of adverse physiological responses. These responses include disruption of the intestinal immune barrier, elevation of inflammatory markers, and diminution of antioxidant capacity [23]. These perturbations collectively impair the efficiency of nutrient digestion and absorption. Therefore, they negatively impact overall growth and health [24]. PQQ modulates inflammatory cytokine expression, exerting anti-inflammatory effects [25]. In this study, the addition of PQQ effectively inhibited the inflammatory factors (IL-1β, IL-6, and TNF-α) in the serum of weaned piglets. In recent research, PQQ downregulates pro-inflammatory cytokines like IL-2, IL-6, TNF-α, and cyclooxygenase-2 (COX-2). This modulation is achieved via the deacetylation activity of SIRT1, which inhibits the NF-κB signaling pathway [2]. Fibroblasts pretreated with pyrroloquinoline quinone (PQQ) exhibit less damage induced by TNF-α [26]. In addition, PQQ can inhibit the expression of pro-inflammatory factors induced by LPS in cell experiments [27]. Additionally, PQQ can enhance the antioxidant capacity of piglets, thereby exerting a protective effect on tissues. This study demonstrated that dietary addition with 4 mg/kg PQQ significantly enhanced the antioxidant capacity of weaned piglets. Previous studies have demonstrated that PQQ addition in feed increases the levels of antioxidant enzymes such as SOD and GSH-Px in the serum of piglets [6]. These are consistent with the results of this study. In laying hens, it has been shown that PQQ addition can improve egg white quality and Haugh unit values. These effects are attributed to PQQ’s antioxidant properties [28]. Studies on HK-2 cells have demonstrated that the protective effects of PQQ are associated with increased levels of antioxidant enzymes [29]. The immune capacity of piglets is also affected by weaning stress [30]. Serum immunoglobulin levels are key indicators of immune function [31]. This study shows that PQQ can increase the levels of IgG and IgA in the serum. Research findings indicate that the addition of PQQ in the diet of piglets is capable of elevating the levels of IgM and IgA in their serum [32]. In addition, a study has shown that PQQ can also modulate the immune response in rats suffering from enteritis [33].

The intestinal barrier is a crucial safeguard for piglets during weaning stress [1]. Analyses of correlations were carried out between the gut microbiota and immune and inflammation-related indicators. As a result, several strains were identified that may modulate immunity and inflammation. However, no statistically significant differences were detected in the relative abundance of these strains across different treatment groups. Based on these findings, based on these findings, we hypothesize that PQQ can affect the relative abundance of intestinal microbiota, but the affected intestinal bacteria are unable to directly influence the host’s immune function. To this end, our focus is directed toward the biological barrier of the intestinal barrier. The biological barrier is composed of intestinal microorganisms. The intestinal microbiota interacts with the host, regulating immunity and metabolism [34]. The biological barrier plays an important role in host nutrient metabolism [35]. This study shows that PQQ can increase gut microbiota abundance and alter the gut microbiota structure of piglets. The differences in *Subdoligranulum* and *Escherichia-Shigella* are due to abnormalities in the individual’s gut microbiota. Adding with PQQ can significantly reduce the relative abundance of *Ruminococcus*. Studies have shown that some strains of *Ruminococcus* can degrade intestinal mucins. As a consequence of this degradation, the barrier function is disrupted, ultimately leading to increased intestinal permeability [36]. Some studies have also shown that *Ruminococcus. gnavus* can induce the production of cytokines such as IL-1β, IL-6, and TNF-α [37]. Given these findings, it is plausible to hypothesize that PQQ may exert a regulatory effect on the gut microbiota by downregulating the relative abundance of *Ruminococcus*. This regulatory effect contributes to the maintenance of intestinal barrier integrity. This study has demonstrated that PQQ can upregulate the abundance of specific bacterial taxa, including *Olsenella*, *norank_f_Muribaculaceae*, *Senegalimassilia*, *Lachnospira*, and *Enterorhabdus*. These taxa are characterized by their enzymatic activities in catalyzing decarboxylation reactions [38]. These activities contribute to cellulose degradation [39], carbohydrate metabolism, and the production of SCFAs [40]. Collectively, these metabolic capabilities are crucial for maintaining intestinal barrier integrity and promoting gut microbiota homeostasis. In addition, this study demonstrates that PQQ can significantly elevate the levels of SCFAs in the piglet gut. Based on correlation analyses between gut microbiota and SCFAs levels, combined with Multiple-Group Comparative Analysis of Species Differences, it is hypothesized that PQQ modulates SCFA concentrations. This modulation is achieved by reducing the abundance of *Ruminococcus* and increasing the abundance of *norank_f_Muribaculaceae*. SCFAs serve as an important indicator of gut health. They are known to play a crucial role in regulating mucosal immunity of intestinal epithelial cells [41].

When administered as a dietary additive, PQQ can modulate mitochondrial energy metabolism in mice. Moreover, it can also mitigate obesity-related metabolic disorders in them [42]. Additionally, studies have demonstrated that dietary addition with PQQ can regulate glycolipid metabolism in weaned piglets by inhibiting the phosphorylation of AMPK [43,44]. Based on this, we focused on the effects of PQQ on the metabolism of weaned piglets. Metabolomics analyses demonstrate that PQQ induces distinct metabolic profiles. Notably, the most pronounced upregulation is observed in the TCA cycle. The TCA cycle is a critical enzyme-catalyzed metabolic pathway. It occurs in the mitochondrial matrix. And it provides essential high-energy substrates for the electron transport chain [21]. Accordingly, PQQ promotes mitochondrial function. Mitochondria maintain the normal physiological functions of lymphocytes by regulating energy metabolism. When immune cells are activated, the metabolic level increases accordingly to meet functional demands such as immune responses [45]. It has also been found that mitochondrial DNA (mtDNA) controls the flux of TCA cycle by maintaining oxidative phosphorylation (OxPhos), which is of great significance for the metabolism and function of B cells [46]. This could serve as a potential mechanism by which PQQ promotes immunoglobulin secretion in weaned piglets. Metabolite analysis revealed that the addition of PQQ can significantly increase the levels of citric acid, isocitric acid and malic acid in the TCA cycle. Empirical evidence has demonstrated that citric acid possesses the capacity to scavenge free radicals [47]. This is consistent with the findings that PQQ enhances antioxidant capacity. The observed increases in isocitric acid and malic acid levels suggest that PQQ may influence hydration reactions within the TCA cycle. However, the precise mechanisms through which PQQ exerts this influence remain to be elucidated. PQQ also upregulates the glucagon signaling pathway to modulate glucose and lipid metabolism in weaned piglets. Studies have demonstrated that PQQ exerts regulatory effects on lipid and energy metabolism in rats [48]. Furthermore, PQQ has been shown to modulate a diverse array of metabolic pathways, including the Phospholipase D signaling pathway, Taste transduction, Sphingolipid signaling pathway, Glycine, serine and threonine metabolism, Neuroactive ligand-receptor interaction, and Arginine and proline metabolism. These findings suggest that PQQ possesses neuroregulatory [49] and amino acid metabolic functions [50].

Given that the focus of this experiment is more on production applications, with greater emphasis on the practical application effects of PQQ in piglets, this study lacks in-depth follow-up research on the later growth and development of piglets. In addition, relevant research methods such as microbial sequencing and metabolic determination are relatively traditional. In future related studies, we will further expand the scope of research, including long-term tracking and comprehensive exploration using more advanced technologies.

## 5. Conclusions

In conclusion, under the experimental conditions employed herein, the incorporation of 4 mg/kg PQQ into the diet may be suggestive of potential multifaceted beneficial effects on weaned piglets. Specifically, dietary PQQ addition appears to enhance immune competence and antioxidant capacity in weaned piglets, a phenomenon that could be associated with modulations in gut microbiota composition and upregulation of the TCA cycle, albeit requiring further validation. These observed metabolic and microbiological alterations tend to collectively contribute to reductions in inflammatory markers and improvements in growth performance parameters in weaned piglets. It suggests that PQQ may exert a potential regulatory role in weaning stress of weaned piglets.

## Figures and Tables

**Table 1:** Composition and nutrient levels of basal diet (air-dry basis, %).

Item	Content
Ingredients	
Corn	67.2
Expanded soybean	7
Expanded soybean meal (CP 43%)	13.6
Fermented soybean meal	5.4
Whey powder	0.8
Imported fish meal (CP 65%)	1.2
Rice husk powder	0.25
Dicalcium hydrogen phosphate	1.12
Limestone powder	0.96
Salt	0.34
Choline chloride	0.1
Lys (78%)	0.56
Met (98%)	0.2
Thr (98%)	0.2
Try	0.07
Trace element premix ^1^	0.5
Vitamin premix ^1^	0.5
Total	100
Nutritional components ^2^	
OM (On an air-dry diet basis)	93.68
CP	19.27
DE (MJ/kg)	14.1
NDF	8.32
ADF	3.81
Lys	1.3
Met + Cys	0.78
Thr	0.86
Ca	0.78
TP	0.61

^1^ The premix provides the following per kilogram of the daily diet: Zinc (Zn), 70 mg (in the form of zinc sulfate); Copper (Cu), 25 mg (in the form of copper sulfate); Manganese (Mn), 20 mg (in the form of manganese sulfate); Iodine (I), 0.4 mg (in the form of calcium iodate); Selenium (Se), 0.4 mg (in the form of sodium selenite). Vitamin A, 7500 IU; Vitamin D3, 750 IU; Vitamin E, 25 IU; Vitamin K3, 2.5 mg; Vitamin B1, 2.0 mg; Vitamin B2, 4.0 mg; Vitamin B6, 10.0 mg; Vitamin B12, 0.025 mg; Niacin, 40 mg; Pantothenic acid, 16.0 mg; Folic acid, 2.0 mg; Biotin, 0.18 mg; Phytase (5000 U/g), 100 mg. ^2^ The Lys, Met + Cys, Thr components are calculated values, and the CP, DE, NDF, ADF, Ca, TP components are the measured values. Lys = Lysine, Met = Methionine, Thr = Threonine, Try = Tryptophan, OM = Organic Matter, CP = Crude Protein, DE = Digestible Energy, NDF = Neutral Detergent Fiber, ADF = Acid Detergent Fiber, Ca = Calcium, TP = Total Phosphorus.

**Table 2:** Performance of weaned pigs fed graded levels of PQQ for 28 d ^1^.

Items	PQQ, mg/kg						*p*-Value		
	0	1	2	4	8	16	T	L	Q
Weight (kg)									
0 d	8.01 ± 0.034	7.97 ± 0.041	7.97 ± 0.027	7.98 ± 0.050	7.97 ± 0.054	7.99 ± 0.067	0.993	0.999	0.901
14 d	11.07 ± 0.054	11.10 ± 0.084	11.24 ± 0.079	11.41 ± 0.103	11.37 ± 0.091	11.28 ± 0.083	0.182	0.185	0.039
28 d	15.92 ± 0.271 ^b^	16.21 ± 0.343 ^b^	16.55 ± 0.098 ^ab^	17.39 ± 0.051 ^a^	17.38 ± 0.175 ^a^	16.93 ± 0.149 ^ab^	0.001	0.021	0.01
ADG (g/d)									
0–14 d	219 ± 4.7	224 ± 4.6	233 ± 5.7	245 ± 4.4	243 ± 2.8	235 ± 1.3	0.191	0.212	0.037
15–28 d	347 ± 5.3 ^b^	365 ± 7.4 ^ab^	380 ± 5.5 ^ab^	427 ± 4.9 ^a^	430 ± 4.2 ^a^	404 ± 7.9 ^ab^	0.002	0.022	0.001
0–28 d	283 ± 4.7 ^b^	295 ± 4.1 ^ab^	306 ± 5.4 ^ab^	336 ± 5.8 ^a^	336 ± 5.7 ^a^	319 ± 4.2 ^ab^	0.001	0.024	0.003
ADFI (g/d)									
0–14 d	453 ± 3.4	451 ± 5.6	452 ± 5.2	443 ± 3.1	441 ± 2.8	439 ± 3.9	0.884	0.235	0.428
15–28 d	755 ± 12.5	801 ± 10.6	831 ± 10.2	781 ± 10.8	799 ± 12.6	777 ± 13.6	0.541	0.804	0.707
0–28 d	604 ± 5.8	626 ± 6.7	642 ± 7.2	612 ± 4.3	620 ± 4.9	608 ± 5.3	0.593	0.55	0.721
F: G									
0–14 d	2.07 ± 0.048	2.03 ± 0.051	1.94 ± 0.037	1.82 ± 0.029	1.83 ± 0.021	1.88 ± 0.051	0.051	0.054	0.006
15–28 d	2.19 ± 0.021 ^a^	2.21 ± 0.030 ^ab^	2.20 ± 0.071 ^ab^	1.84 ± 0.063 ^b^	1.88 ± 0.027 ^b^	1.93 ± 0.034 ^ab^	0.02	0.023	0.006
0–28 d	2.14 ± 0.042 ^a^	2.13 ± 0.031 ^a^	2.10 ± 0.029 ^ab^	1.83 ± 0.041 ^b^	1.86 ± 0.033 ^b^	1.91 ± 0.028 ^ab^	0.005	0.013	0.001

^1^ Values are means of pigs per treatment (*n* = 6). a, b, means within a row without common superscripts differ significantly (*p* < 0.05). T = treatment; L = linear; Q = quadratic, ADG = average daily gain; ADFI = average daily feed intake; F: G = feed conversion ratio (feed: gain).

**Table 3:** Hematology of weaned piglets fed graded levels of PQQ for 28 d ^1^.

Items	PQQ, mg/kg						*p*-Value		
	0	1	2	4	8	16	T	L	Q
14 d									
WBC (10^9^/L)	20.06 ± 0.521	20.55 ± 0.375	18.89 ± 0.322	20.93 ± 0.398	21.14 ± 0.541	22.32 ± 0.635	0.198	0.071	0.083
RBC (10^12^/L)	6.22 ± 0.042	6.39 ± 0.032	6.20 ± 0.037	6.12 ± 0.025	6.11 ± 0.020	6.36 ± 0.045	0.100	0.566	0.056
HGB (g/L)	95.42 ± 0.687	93.67 ± 0.591	96.17 ± 0.575	92.42 ± 0.513	92.75 ± 0.589	93.00 ± 0.879	0.436	0.228	0.3
HCT (%)	34.67 ± 0.442	32.97 ± 0.087	34.09 ± 0.413	33.12 ± 0.162	33.92 ± 0.362	34.11 ± 0.171	0.262	0.743	0.617
MCV (fl)	54.54 ± 0.379	55.68 ± 0.219	54.09 ± 0.232	55.30 ± 0.317	54.56 ± 0.198	54.28 ± 0.199	0.519	0.44	0.721
MCH (pg)	15.40 ± 0.050	14.90 ± 0.041	15.26 ± 0.047	15.44 ± 0.083	15.38 ± 0.054	15.21 ± 0.624	0.312	0.885	0.621
MCHC (g/L)	283.4 ± 0.85	284.3 ± 0.97	283.6 ± 0.62	281.3 ± 0.65	283.4 ± 0.62	282.8 ± 0.67	0.838	0.671	0.833
RDW (%)	20.64 ± 0.784	19.48 ± 0.079	20.14 ± 0.125	20.13 ± 0.176	20.00 ± 0.264	20.10 ± 0.146	0.083	0.856	0.804
PLT (10^9^/L)	531.8 ± 9.89	547.7 ± 9.72	503.3 ± 8.54	564.8 ± 11.63	566.4 ± 11.97	490.0 ± 7.96	0.095	0.245	0.055
28 d									
WBC (10^9^/L)	21.10 ± 0.378	20.58 ± 0.746	19.70 ± 0.248	20.13 ± 0.211	19.03 ± 0.247	19.23 ± 0.263	0.310	0.059	0.078
RBC (10^12^/L)	6.09 ± 0.043	6.05 ± 0.041	5.93 ± 0.051	5.80 ± 0.058	5.87 ± 0.076	5.98 ± 0.062	0.106	0.45	0.071
HGB (g/L)	94.92 ± 0.468	93.00 ± 0.547	92.50 ± 0.613	92.17 ± 0.546	93.00 ± 0.945	92.50 ± 0.871	0.584	0.402	0.48
HCT (%)	32.47 ± 0.632	31.33 ± 0.431	31.68 ± 0.489	31.50 ± 0.178	31.64 ± 0.174	31.11 ± 0.368	0.225	0.104	0.251
MCV (fl)	52.45 ± 0.538	52.18 ± 0.274	52.59 ± 0.387	52.68 ± 0.346	53.92 ± 0.145	53.78 ± 0.201	0.112	0.071	0.063
MCH (pg)	15.53 ± 0.054	15.64 ± 0.046	15.47 ± 0.033	15.45 ± 0.065	15.93 ± 0.076	15.78 ± 0.054	0.163	0.076	0.164
MCHC (g/L)	295.1 ± 1.75	296.3 ± 0.98	293.0 ± 0.84	294.7 ± 1.26	292.1 ± 0.84	291.2 ± 0.96	0.260	0.032	0.091
RDW (%)	18.52 ± 0.221	18.61 ± 0.537	19.34 ± 0.487	18.48 ± 0.337	18.58 ± 0.429	18.95 ± 0.210	0.148	0.591	0.751
PLT (10^9^/L)	441.7 ± 8.98	445.0 ± 6.87	431.7 ± 5.78	474.2 ± 5.02	407.8 ± 6.34	406.4 ± 8.52	0.204	0.081	0.217

^1^ Values are means of pigs per treatment (*n* = 6). Means within a row without common superscripts differ significantly (*p* < 0.05). T = treatment; L = linear; Q = quadratic, WBC = White Blood Cell Count, RBC = Red Blood Cell Count, RDW = Red Cell Distribution Width, HCT = Hematocrit, MCV = Mean Corpuscular Volume, MCH = Mean Corpuscular Hemoglobin, MCHC = Mean corpuscular hemoglobin concentration, HGB = Hemoglobin, PLT = Platelet Count.

**Table 4:** Immunity and antioxidation of piglets fed graded levels of PQQ for 28 d ^1^.

Items	PQQ, mg/kg			*p*-Value		
	0	4	8	T	L	Q
14 d						
IgA (g/L)	2.18 ± 0.058	2.62 ± 0.075	2.28 ± 0.042	0.167	0.760	0.166
IgG (g/L)	17.05 ± 0.952 ^b^	19.06 ± 0.625 ^a^	19.35 ± 0.739 ^a^	0.002	0.010	0.010
IgM (g/L)	1.36 ± 0.051	1.47 ± 0.023	1.45 ± 0.043	0.624	0.536	0.177
T-AOC (U/mL)	7.61 ± 0.537 ^b^	8.50 ± 0.424 ^a^	8.33 ± 0.571 ^ab^	0.004	0.228	0.040
SOD (U/mL)	71.73 ± 1.462 ^b^	80.96 ± 1.205 ^a^	76.50 ± 1.168 ^ab^	0.003	0.274	0.115
GSH-Px (μmol/L)	127.61 ± 0.279 ^b^	140.74 ± 0.254 ^a^	134.00 ± 0.213 ^ab^	0.001	0.960	0.965
TNF-α (ng/mL)	51.72 ± 0.783 ^a^	44.35 ± 0.742 ^b^	43.75 ± 0.751 ^b^	0.001	0.026	0.150
IL-1β (ng/mL)	23.30 ± 0.328 ^a^	19.36 ± 0.375 ^b^	18.05 ± 0.312 ^b^	0.001	0.020	0.010
IL-6 (ng/mL)	145.26 ± 1.53 ^a^	121.10 ± 1.24 ^b^	115.83 ± 1.87 ^c^	0.001	0.002	0.020
28 d						
IgA (g/L)	2.22 ± 0.258 ^b^	2.80 ± 0.236 ^a^	2.60 ± 0.109 ^a^	0.001	0.023	0.010
IgG (g/L)	26.01 ± 0.533	27.24 ± 0.418	27.41 ± 0.454	0.332	0.172	0.332
IgM (g/L)	1.38 ± 0.037	1.50 ± 0.028	1.47 ± 0.030	0.624	0.555	0.627
T-AOC (U/mL)	10.21 ± 0.688 ^b^	12.39 ± 0.745 ^a^	11.12 ± 0.636 ^b^	0.001	0.111	0.010
SOD (U/mL)	90.53 ± 0.845	93.14 ± 0.746	90.53 ± 0.907	0.221	1.000	0.221
GSH-Px (μmol/L)	163.88 ± 0.754	166.76 ± 0.622	167.32 ± 0.619	0.480	0.256	0.479
TNF-α (ng/mL)	38.74 ± 0.385	40.10 ± 0.527	38.42 ± 0.372	0.187	0.296	0.157
IL-1β (ng/mL)	17.66 ± 0.276	17.08 ± 0.257	17.61 ± 0.293	0.159	0.718	0.294
IL-6 (ng/mL)	121.14 ± 0.951 ^a^	104.75 ± 0.924 ^b^	93.67 ± 0.964 ^c^	0.001	0.010	0.010

^1^ Values are means SEM of pigs per treatment (*n* = 6). a, b, c, means within a row without common superscripts differ significantly (*p* < 0.05). T = treatment; L = linear; Q = quadratic, IgG = Immunoglobulin G, IgA = Immunoglobulin A, IgM = Immunoglobulin M, GSH-Px = Glutathione Peroxidase, SOD = Superoxide Dismutase, T-AOC = Total Antioxidant Capacity, TNF-α = Tumor Necrosis Factor-α, IL-1β = Interleukin-1β, IL-6 = Interleukin-6.

**Table 5:** Intestinal villus length and crypt depth of piglets fed graded levels of PQQ for 28 d ^1^.

Items	PQQ, mg/kg			*p*-Value		
	0	4	8	T	L	Q
Duodenum						
Villus length (μm)	443.08 ± 15.917	422.00 ± 6.949	449.94 ± 8.341	0.214	0.379	0.531
Crypt depth (μm)	467.12 ± 15.206	427.02 ± 15.715	453.18 ± 26.422	0.372	0.824	0.179
V/C	0.96 ± 0.061	1.00 ± 0.044	1.01 ± 0.061	0.794	0.635	0.249
Jejunum						
Villus length (μm)	387.35 ± 10.931	363.52 ± 16.905	378.79 ± 13.042	0.485	0.571	0.397
Crypt depth (μm)	307.27 ± 20.922	278.60 ± 10.410	324.71 ± 9.247	0.109	0.847	0.361
V/C	1.28 ± 0.065	1.31 ± 0.075	1.17 ± 0.065	0.349	0.557	0.609
Jejunum						
Villus length (μm)	313.60 ± 19.837	268.08 ± 16.218	347.75 ± 28.999	0.071	0.497	0.864
Crypt depth (μm)	328.23 ± 13.086	274.58 ± 21.909	305.92 ± 27.073	0.240	0.656	0.491
V/C	0.96 ± 0.067	0.98 ± 0.047	1.15 ± 0.081	0.118	0.315	0.530

^1^ Values are means SEM of pigs per treatment (*n* = 6). Means within a row without common superscripts differ significantly (*p* < 0.05). T = treatment; L = linear; Q = quadratic, V/C = villus length (μm)/crypt depth (μm).

**Table 6:** Short-Chain fatty acid of piglets fed graded levels of PQQ for 28 d ^1^.

Items	PQQ, mg/kg			*p*-Value		
	0	4	8	T	L	Q
Acetic Acid (mg/g)	16.64 ± 1.477 ^b^	50.95 ± 2.754 ^a^	94.56 ± 3.575 ^a^	0.001	<0.01	0.020
Propionic Acid (mg/g)	9.16 ± 0.981 ^c^	40.25 ± 2.554 ^b^	117.32 ± 4.792 ^a^	0.001	<0.01	0.347
Isobutyric Acid (mg/g)	0.027 ± 0.002 ^c^	0.82 ± 0.143 ^b^	0.89 ± 0.241 ^a^	0.007	<0.01	0.882
Butyrate (mg/g)	5.15 ± 0.438 ^b^	29.64 ± 3.523 ^a^	36.51 ± 3.974 ^a^	0.001	<0.01	0.074
Isovaleric Acid (mg/g)	0.048 ± 0.006 ^b^	0.72 ± 0.213 ^b^	0.83 ± 0.225 ^a^	0.001	<0.01	0.015
Valeric Acid (mg/g)	0.22 ± 0.004 ^c^	0.24 ± 0.087 ^b^	2.97 ± 0.949 ^a^	0.001	<0.01	0.040

^1^ Values are means SEM of pigs per treatment (*n* = 6). a, b, c, means within a row without common superscripts differ significantly (*p* < 0.05). T = treatment; L = linear; Q = quadratic.

## Data Availability

The DNA sequences of this article were deposited in the National Center for Biotechnology Information (NCBI) Sequence Read Archive (SRA) repository under accession number PRJNA1232724.
